# Contemporary Synthetic Glycoscience: A Theme Issue Dedicated to the Memory of Hans Paulsen

**DOI:** 10.3390/molecules30234617

**Published:** 2025-12-01

**Authors:** Joachim Thiem

**Affiliations:** Institute of Organic Chemistry, Department of Chemistry, Faculty of Science, University of Hamburg, Martin-Luther-King-Platz 6, D-20146 Hamburg, Germany; joachim.thiem@uni-hamburg.de

Annually, the biomass of the Earth, estimated to be about 200 billion tons, is nourished by photosynthesis and degraded via various pathways. The primary product, glucose, is transformed into a huge number of distinct and structurally complex mono-, di-, tri-, and oligosaccharides, which provide glyco-based molecules for nourishment, as well as structural units. Together, these constitute about 95% of the total biomass, and the remaining 5% is transformed into all the other organic components involved in Earth’s life-cycles.

In addition, nature has provided an overwhelmingly large number of complex glycosylated components, which underpin the life and health of all animate beings. Thus, fascinating questions involving the operating sequences and mechanisms underpinning all life on Earth have encouraged chemists to synthesize and study increasingly complex glucose derivatives.

One of the dominant figures in this area was Hans Paulsen, based in Hamburg, Germany, who, over his decades-long his career, dedicated himself and his research group to syntheses in carbohydrate chemistry.

This Special Issue compiles the work of former colleagues of Hans Paulsen from around the world, who exchanged ideas with him extensively and contributed seminal studies covering a variety of exciting developments in contemporary glycoscience. In this introduction, I will briefly comment on the studies in synthetic and analytical glycoscience compiled herein.

As has been known for decades, the expression of glycosyltransferases in cancer cells generates tumor-associated carbohydrate antigens (TACAs) [1]. For example, aberrant sialylation on cell surfaces is known to be associated with cancer progression. The over-sialylation of glycoproteins on cancer cells increases the number of interactions with specific sialic acid-binding immunoglobulin-type lectins (Siglecs) on immune cells. This results in the suppression of the immune response against cancer cells, known as immune checkpoint inhibition [2,3]. Wong et al., who have, for a number of decades, been among the leading research groups employing chemo-enzymatic syntheses for highly complex hetero-oligosaccharides [4], contribute a number of convincing scalable chemo-enzymatic approaches to the synthesis of DSGb5 and complex sialylated hetero-oligosaccharides. These sialylated glycans from cancer-associated glycolipids were used by Wong et al. to compose glycan microarrays [5].

Cardiac glycosides [6] have been used for centuries in clinics for the treatment of heart failure and cardiac rhythm disorders [7,8]. Yu et al., who are among the leading research groups devoted to the synthesis of complex steroid glycosides [9], demonstrate the total syntheses of the structurally challenging cardiac glycosides acospectoside A and acovenoside B [10].

Glycosphingolipids (GSLs) in cell membranes function as important regulators of essential biological processes such as cellular recognition and signaling, cell differentiation, and proliferation [11,12,13,14]. Thus, the contribution of Guo et al. focuses on syntheses of β-lactosylceramide (β-LacCer) analogs as key intermediates in the study of complex GSL biosynthetic processes [15].

Brucellosis constitutes a serious zoonotic bacterial—and highly contagious—disease in many animals, including valuable cattle [16,17]. The binding of murine monoclonal antibodies (mAbs) to hexasaccharide fragments of epitopes of the Brucella *O*-antigen is soon to be explored in computational study [18]. Bundle, Wood et al. were able identify stable binding modes, which further our understanding of the recognition mechanism [19]. In the future, designing synthetic glycomimetics and engineering epitope-specific antibodies *en route* to vaccine design might be possible.

Since the original Koenigs–Knorr glycosylation in 1901 (124 years ago [20]), a compelling number of improvements on, and alternatives to, this seminal reaction have published. Almost every scientist working in the synthesis on glycosylated components is required to employ this reaction, and each will be likely to affirm the following statement from Hans Paulsen, made in 1982: *“Although we have now learned to synthesize oligosaccharides, it should be emphasized that each oligosaccharide synthesis remains an independent problem, whose resolution requires considerable systematic research and a good deal of know-how. There are no universal reaction conditions for oligosaccharide syntheses”* [21]. One of the more recent modifications made in chemical glycosylation is the “4K reaction” [22]. The studies of Demchenko et al. demonstrate further improvements to this glycosylation modification, employing iron(III) triflate as an efficient activator of thioglycosides in this reaction pathway [23].

An important issue in glycobiology concerns bacterial adhesion as the initial step of infectious diseases. A closer understanding of the mechanisms involved could be of interest in preventing the adhesion of bacteria, for example, by employing carbohydrate-based inhibitors to facilitate further breakthroughs in diagnostics and therapy [24]. Of particular interest are functional glycomimetics, which are suited to studying the details of carbohydrate recognition, such as the varying distances between multiple glycoligands [25]. In their contribution, Lindhorst et al. employed xylopyranosides as a scaffold for the presentation of two mannoside ligands of the bacterial lectin FimH. The chair conformation of xylosides could be switched between alternative conformations, thus flipping the ligands from a diequatorial into a diaxial position. Further, such conformational switches may function as advanced smart molecular tools with which to study structural binding conditions in carbohydrate recognition [26].

Essential for membrane functions, cell recognition, and the maintenance of the nervous system are gangliosides such as GM2 (GalNAcβ1-4Galβ1-4Glcβ-Cer). Some rare genetic lysosomal storage diseases—e.g., Tay-Sachs disease (TSD) and Sandhoff disease (SD)—occur due to deficient β-*N*-acetyl-hexosaminidase activity, leading to decreased catabolism of β-*N*-acetyl-hexosamine-containing ganglioside GM2 in the lysosomes and, consequently, causing damage to cells and tissues, as well as severe neurological symptoms [27,28]. A number of contemporary studies have aimed to express and purify human B4GALNT1, the enzyme that transfers the GalNAc residue in the β1-4 linkage to the Galβ1-4 residue of the gangliosides GM3, GD3, and other GSLs. A number of studies have focused on characterizing its activity and exploring its structural features via protein modeling and substrate docking. Brockhausen et al., for a complex of B4GALNT1 docked with its donor substrate, UDP-GalNAc, were able determine the relevant amino acids near the docking site that were likely involved in UDP-GalNAc binding and catalysis [29]. Thus, further studies *en route* to drug development, including the kinetics of inhibition, toxicity, and drug delivery to neurons accumulating GM2, are to follow.

Cell membranes are embedded with a glycocalix consisting of a complex mixture of glycoproteins, glycolipids, complex oligosaccharides, glycoconjugates, and proteoglycans. Thus, cellular processes, such as bacterial and viral infection, cancer metastasis, the modulation and activation of the immune system, tissue differentiation and development, and further intercellular recognition events, are controlled by glycoconjugates [30,31,32,33,34]. For mimicking the glycocalyx, the self-assembled monolayer (SAM) formation of carbohydrate derivatives on gold nanoparticles was selected, allowing for the study of carbohydrate–protein and carbohydrate–carbohydrate interactions. The novel modular approach reported here includes facile and rapid syntheses for linking spacers and carbohydrate derivatives, enhancing binding events. Immobilization was performed on biorepulsive aminoxy-substituted gold nanoparticles via the oxime formation of aldehyde-functionalized mono-, di-, and complex trisaccharides. Uniform gold nanoparticles could be obtained, and effective immobilization and binding studies were presented for concanavalin A. Through this novel approach, Thiem et al. revealed a number of advantageous perspectives to be taken on various biomimetic studies of carbohydrates and carbohydrate-based array development for diagnostics and screening [35].

The innate immune system is sensitive to lipopolysaccharides (LPS), which play a pivotal role in immune response [36]. Lipid A, composed of a mono- or di-phosphorylated di-glucosamine core with multiple fatty acid chains, anchors LPS into the bacterial membrane and is largely responsible for the toxicity of Gram-negative bacteria [37]. In their contribution, D’Orazio, Lay et al. present syntheses and a computational evaluation of a library of glycolipid analogues to B. fragilis lipid A, revealing a rationally designed approach to developing novel anti-inflammatory agents [38].

Pathogenic bacteria often utilize cell surface sialylation to evade the host immune system via molecular mimicry of the host sialo-glycoconjugates [39]. Human pathogen Neisseria meningitidis serotype B (NmB) expresses both the sialylated capsule and surface lipooligosaccharides—pivotal virulence factors [40]. In NmB’s sialylation pathway, CMP-sialic acid synthetase (CSS) is essential for sialic acid transfer. Along various routes (compare also e.g., [41]), functionalized derivatives of neuraminic acid β-methyl glycoside (Neuβ2Me) were synthesized as candidates for the inhibition of N. meningitidis CSS. Direct interaction with the enzyme was confirmed by saturation transfer difference (STD) NMR [42,43]. In their contribution, Jennings, Münster-Kühnel, and von Itzstein et al. provide data encouraging the further development of potential inhibitors for the treatment of bacterial meningitis [44].

Nature makes sucrose annually in virtually limitless quantities, the majority of which is consumed via the food industry. Further uses include the production of bioethanol, biodegradable surfactants, and polymers [45]. Application of this cheap raw material with 100% optical purity and eight genuine stereogenic centers for the synthesis of various sophisticated products is of particular interest [46]. In their review, Jaraosz et al. focused on approaches to the use of sucrose in the design of fine chemicals, the macrocyclic scaffold, and further-modified derivatives for various applications [47].

For the colonization of human organs, pathogenic bacteria use a multilayer cell envelope largely composed of glycans and their conjugates [48,49]. Most Gram-negative and some Gram-positive bacteria comprise a capsule largely composed of glycans and their conjugates [50]. Thus, the synthesis of spacer-armed phosphooligosaccharides, structurally related to the capsular phosphoglycans of pathogenic bacteria, is of particular interest (e.g., [51]).

In their extended review, Nifantiev et al. summarize our current understanding of the preparation and immunogenicity of neoglycoconjugates based on synthetic phospho-oligosaccharides to foster prospects for the development of conjugate vaccines on the basis of synthetic phosphooligosaccharide antigens [52].

Acknowledging Hans Paulsen and his collaborators for their seminal study of glycoprotein biosynthesis, the final review by Brockhausen sheds light on their many individual and decisive contributions. The stepwise development is demonstrated via the continual interaction of synthetic and biochemical glycoscientists, resulting in cleverly designed components [53].

This Special Issue contains ten contributions discussing challenging topics pertaining to both syntheses [5,10,15,23,26,38,44] and analyses [19,29]. Additionally, the review articles included discuss the relevant aspects of sucrose-based components [47] and the immunogenicity of neoglycoconjugates [52]. The final feature review describes the numerous synthetic contributions of the Paulsen group to solving questions pertaining to glycoprotein biosynthesis [53]. In this Special Issue, a broad overview of contemporary glycoscience research is compiled, indicating the significant influence of this subject in natural products and heterocyclic chemistry, as well as its value in addressing broader issues in biology and medicine.
